# Bogotá River anthropogenic contamination alters microbial communities and promotes spread of antibiotic resistance genes

**DOI:** 10.1038/s41598-019-48200-6

**Published:** 2019-08-13

**Authors:** Carlos Eduardo Posada-Perlaza, Adán Ramírez-Rojas, Paola Porras, Boahemaa Adu-Oppong, Ana-María Botero-Coy, Félix Hernández, Juan M. Anzola, Lorena Díaz, Gautam Dantas, Alejandro Reyes, María Mercedes Zambrano

**Affiliations:** 1 Computational Biology and Microbial Ecology Group, Department of Biological Sciences, Universidad de los Andes, Bogotá, D.C. Colombia; 2 Max Planck Tandem Group in Computational Biology, Universidad de los Andes, Bogotá, D.C. Colombia; 3 Molecular Genetics and Bioinformatics, Corporación CorpoGen, Bogotá, D.C. Colombia; 4 Molecular Genetics and Antimicrobial Resistance Unit, International Center for Microbial Genomics, Universidad El Bosque, Bogotá, D.C. Colombia; 5 Center for Genome Science and Systems Biology, Washington University School of Medicine, St. Louis, MO USA; 6 Research Institute for Pesticides and Water, University Jaume I, Castellón, Spain; 7 Department of Pathology and Immunology, Washington University School of Medicine, St. Louis, MO USA; 8 Department of Molecular Microbiology, Washington University School of Medicine, St. Louis, MO USA; 9 Department of Biomedical Engineering, Washington University, St. Louis, MO USA

**Keywords:** Metagenomics, Antimicrobial resistance

## Abstract

The increase in antibiotic resistant bacteria has raised global concern regarding the future effectiveness of antibiotics. Human activities that influence microbial communities and environmental resistomes can generate additional risks to human health. In this work, we characterized aquatic microbial communities and their resistomes in samples collected at three sites along the Bogotá River and from wastewaters at three city hospitals, and investigated community profiles and antibiotic resistance genes (ARGs) as a function of anthropogenic contamination. The presence of antibiotics and other commonly used drugs increased in locations highly impacted by human activities, while the diverse microbial communities varied among sites and sampling times, separating upstream river samples from more contaminated hospital and river samples. Clinically relevant antibiotic resistant pathogens and ARGs were more abundant in contaminated water samples. Tracking of resistant determinants to upstream river waters and city sources suggested that human activities foster the spread of ARGs, some of which were co-localized with mobile genetic elements in assembled metagenomic contigs. Human contamination of this water ecosystem changed both community structure and environmental resistomes that can pose a risk to human health.

## Introduction

It is now well recognized that the medical use of an antibiotic is inevitably followed by the appearance of resistant bacterial strains. The rise in antibiotic resistance worldwide and the menace posed by antibiotic resistant pathogens to human health have led to recent global action plans to tackle antimicrobial resistance^1^. Bacterial pathogens resistant to one or more antibiotics, and even to all known drug regimens, as is the case of XDR-TB^2^, can undermine treatments and lead to increased health care costs due to prolonged illness and hospital stays^1,3^. Resistant strains are responsible for a great number of deaths worldwide, with an annual death toll estimated to rise to 10 million people by 2050^4^. The current situation is aggravated by the slow rate at which new antimicrobial drugs are being developed and by the spread of antibiotic resistance genes (ARGs) both in the community and in settings where microorganisms are exposed to the selective pressure of antibiotics, such as hospitals.

Recent studies using mostly metagenomic approaches have shown that ARGs are present in various natural environments, even those considered pristine or ancient^5–7^. These environmental resistomes are not necessarily linked to human activities and the modern use of antibiotics; rather, they originate from years of evolution as microbes interact and adapt to survive in variable environmental conditions. Microorganisms also have the unique capacity to exchange genetic material by means of mobile genetic elements, which give them greater plasticity and the possibility of accessing the genetic pool of other microbes^8^. Environmental resistomes are extensive and diverse and have gained interest because they may serve as reservoirs for ARGs that could be potentially transferred to clinically relevant bacterial pathogens.

Human activities, recognized as one of the main drivers of evolution in the planet^9^, have transformed various environments by introducing contaminants and compounds that can adversely affect ecosystems and microbial communities^10^. Chemicals, toxic metals and antibiotics can accumulate in the environment due to overuse or unregulated waste management practices, and can inadvertently promote the appearance of resistant bacteria^11^. Certain activities, such as agricultural spread of manure and sewage disposal can introduce ARGs and resistant microorganisms into the environment and create new opportunities for interaction among bacteria of human, animal and environmental origin^8^. Although aquatic environments are particularly important because they provide a basic resource, they receive effluents from industrial and human activities, and thus they represent a unique setting for the acquisition and spread of ARGs^12^, as well as for the proliferation of resistant bacteria^13,14^.

Several studies have looked at the influence of human activities on the presence of antimicrobial agents, resistant bacteria and ARGs in freshwater ecosystems. Antibiotics released into the environment can persist and continue to exert selective pressure for resistance determinants^15,16^, perhaps even at concentrations that can fall below minimal inhibitory doses^17^. Wastewater treatment plants, which can reduce bacterial and ARG load, can still serve as reservoirs of resistant bacteria and ARGs that may impact bacterial water communities^18–20^. Other human activities and anthropogenic pollutants, such as animal feeding and industrial and pharmaceutical wastes, have also been shown to contribute to ARG abundance and dissemination in rivers^21^ and in estuary sediments^22^. The recent use of shotgun metagenomics showed that wastewater contamination of river waters resulted in increased abundances of ARGs, mobile genetic elements and bacterial genera known to harbor pathogenic species^23^. A study of several lakes also found that ARGs were present even in less impacted locations, suggesting a role of these freshwater reservoirs in the spread of resistance markers^24^. In fact, a recent meta-analysis of lakes indicated that contamination with antibiotics and ARGs occurs on a global scale^14^. Overall these studies show that human activities directly affect both microbial communities and resistance genes in freshwater ecosystems, stressing the importance of efficient waste and pollutant management to limit inadvertent effects on the environment and humans.

The surveillance and study of microorganisms in impacted environments can provide information on how microbial communities and ARGs change due to human activities. ARGs have also been proposed as a proxy for understanding anthropogenic influence on dispersion of microorganisms and their genes in the environment^10^. Knowledge of these environmental resistomes and their possible role in human health can raise awareness and help to catalyze actions to control contamination and potential risks. In this work, we studied aquatic microbial communities and antibiotic resistance markers in the Bogotá River, a vital freshwater supply for the region that is greatly deteriorated by industrial, agricultural and municipal discharges before, during and after its passage through the city of Bogotá^25^. A variety of pharmaceutical compounds, including antibiotics, are also commonly found in effluent wastewater and surface waters from Bogotá^26^. However, work is lacking regarding the effect of pollutants on the microbial communities and the ARGs present in this river ecosystem. This work shows that anthropogenic contamination alters the structure and diversity of aquatic microbial communities that in turn can provide a setting for microbial interactions and dispersion of resistance determinants in the environment.

## Results

### Human activities adversely affect water quality

**Figure 1:**
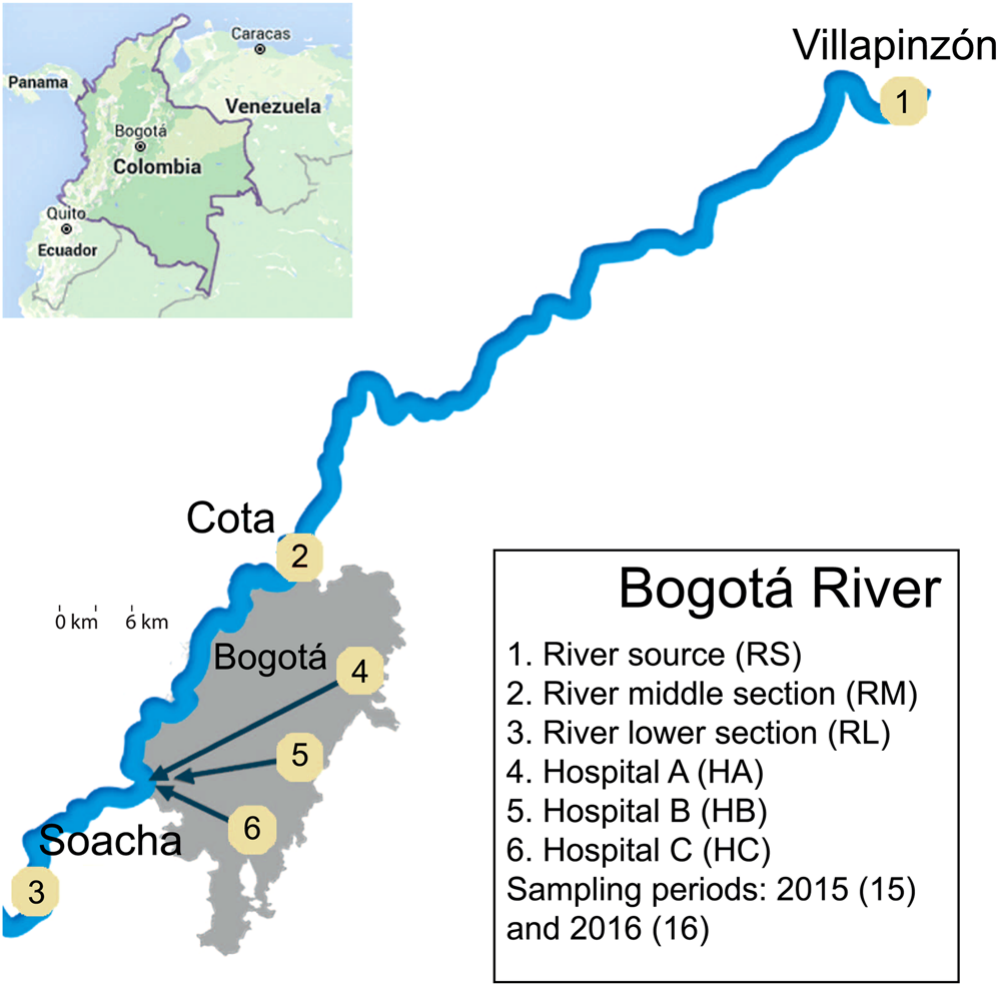
Location of sampling sites. Map highlighting the Bogotá river course and sites for sample collection at the origin (1), before (2) and after (3) Bogotá. Relative locations of three hospitals are shown (4–6) in Bogotá, where samples were also obtained. Inset, the location of Bogotá in Colombia.

Samples with distinct levels of anthropogenic influence were collected from the Bogotá River (river source (RS), middle section (RM) and lower section (RL), in increasing order of anthropogenic influence) and from wastewater effluents at three hospitals in Bogotá (hospitals A (HA), B (HB) and C (HC)) (Fig. 1). Triplicate samples used for physicochemical analyses indicated higher levels of contamination in samples from RL and hospitals, seen particularly in the higher biological oxygen demand (BOD) and chemical oxygen demand (COD) values, when compared to those of RS (Table 1). Duplicate samples were analyzed by UHPLC-MS/MS for the presence of nine antibiotics and seven commonly used pharmaceutical compounds (Supplementary Methods). While all the 16 evaluated compounds were undetectable or present at very low concentrations (below 0.02 µg/l) in the RS sample, except for ciprofloxacin and norfloxacin (Table 1, Supplementary Table S1), they were present in the RL sample at higher concentrations than the other river samples (RS and RM) in 15 out of 16 cases. The three hospital wastewater samples contained more antibiotics than river samples and, in a few cases, concentrations were >5 µg/L, more than double what was observed in river waters, specifically for norfloxacin, sulfamethoxazole, trimethoprim, ciprofloxacin and clarithromycin (Table 1). Other drugs, such as painkillers and medication for high blood pressure, were also detected in the river and hospital samples (Supplementary Table S1). The presence of these pharmaceutical compounds is indicative of the anthropogenic influence on these water systems.

**Table 1:** Physicochemical parameters and antibiotic concentrations of water samples.

Sample^a^	Physicochemical parameter^b^				Antibiotic concentration (µg/L)^c^								
	BOD (mgO_2_/L)	COD (mgO_2_/L)	TSS (mg/L)	TN (%)	MNZ	SMX	TMP	NOR	CIP	CLI	CLR	ERY	AZM
RS15	<21.3	19	0.25	5.2	ND	0.02	ND	2.03	0.57	ND	ND	0.01	ND
RM15	57	96	28	13	0.18	0.17	0.05	2.03	0.54	0.02	0.47	0.01	0.56
RL15	247.1	433	100	12	0.33	1.40	0.29	2.12	0.68	0.01	0.93	0.05	0.99
HA15-1	286	525	131	69	0.36	0.06	0.04	2.43	0.70	0.69	ND	0.01	ND
HA15-2	483	895	109	41	0.36	14.56	7.10	63.22	0.86	0.60	ND	0.03	0.50
HA15-3^d^	243	505	90	52									
HB15-1	<21.3	33	136	32	0.97	3.73	11.20	10.92	2.06	7.22	1.59	0.05	1.47
HB15-2	199	368	103	59	9.00	2.17	1.51	3.47	9.98	0.31	0.73	0.03	0.82
HB15-3^d^	322	577	81	52									
HC15-1	444	847	81	56	9.08	9.20	1.63	6.01	3.06	4.23	17.53	ND	10.57
HC15-2	243	528	63	68	7.61	3.66	0.94	3.83	8.59	4.87	1.71	ND	1.62
HC15-3^d^	470	729	124	35									

^a^Samples collected in 2015 from the Bogotá River source (RS), middle section (RM) or lower section (RL), and from hospitals (HA, HB, HC; 3 samples each).

^b^BOD, biological oxygen demand; COD, chemical oxygen demand; TSS, total suspended solids; TN, total nitrogen.

^c^MNZ, metronidazole; SMX, sulfamethoxazole; TMP, trimethoprim; NOR, norfloxacin; CIP, ciprofloxacin; CLI, clindamycin; CLR, clarithromycin; ERY, erythromycin; AZM, azithromycin; ND, not detected.

^d^Samples HA15-3, HB15-3, HC15-3 were not analyzed for antibiotics.

The microbiological quality of the sampled water was assessed by estimating total coliforms and *Escherichia coli* cells, common indicators of fecal contamination. There was low recovery from the RS samples, consistent with previous reports for the Bogotá River^27^, while the RM, RL and hospital samples had similar *E. coli* counts (between 10^6^ and 10^8^ CFU/L) and total counts (between 10^7^ to 10^8^ CFU/L) (Table 2). These high bacterial loads in the RM and RL sites are indicative of poor water quality and of possible discharges into the river with deficient or no prior treatment. Isolates were recovered from water samples plated on chromogenic selective indicator media containing antibiotics only from the RL samples: *Klebsiella pneumoniae* on CARBA agar for detection of carbapenemase producing Enterobacteriaceae, *K. pneumoniae* and *E. coli* on agar selective for extended-spectrum β-lactamases, and methicillin-resistant, coagulase-negative Staphylococci. In hospital A, we confirmed growth of two vancomycin-resistant isolates (*Enterococcus faecium* VanA) and one *K. pneumoniae* on CARBA agar. No counts were conducted since these microorganisms were expected to be present at very low levels.

**Table 2:** Bacterial counts in Chromocult® Coliform Agar.

Sample^a^	Bacterial counts (CFU/L)			
	Total	Coliforms	*E. coli*	Other^b^
RS15-1	7E + 03	3E + 03	0E + 00	4E + 03
RS15-2	3E + 04	7E + 03	7E + 02	2E + 04
RS16	2E + 05	2E + 04	1E + 03	1E + 05
RM15-1	7E + 07	2E + 07	3E + 06	4E + 07
RM15-2	2E + 08	2E + 08	2E + 07	9E + 06
RM16	1E + 08	8E + 07	4E + 06	6E + 07
RL15-1	7E + 07	2E + 07	4E + 07	1E + 07
RL15-2	4E + 08	2E + 08	2E + 08	1E + 08
RL16	2E + 09	3E + 08	8E + 07	1E + 09
HA15-1	3E + 08	2E + 08	5E + 06	8E + 07
HA15-2	6E + 07	3E + 07	9E + 06	3E + 07
HA15-3	3E + 08	2E + 08	1E + 08	3E + 07
HA16	3E + 08	1E + 08	1E + 08	4E + 07
HB15-1	2E + 08	6E + 07	4E + 07	6E + 07
HB15-2	3E + 08	1E + 07	2E + 08	8E + 06
HB15-3	2E + 08	8E + 07	4E + 07	4E + 07
HB16	1E + 08	2E + 07	4E + 07	4E + 07
HC15-1	5E + 08	5E + 08	1E + 06	6E + 07
HC15-2	9E + 07	4E + 07	3E + 07	2E + 07
HC15-3	2E + 08	6E + 07	5E + 07	5E + 07
HC16	1E + 07	7E + 06	3E + 05	2E + 06

^a^Sample names as described in Table 1.

^b^Other Gram-negative bacteria.

Both physicochemical and microbiological water quality results, as well as the presence of pharmaceutical compounds, indicated increasing contamination in rural and urban areas (RM, RL), consistent with previous reports of water quality deterioration^25^, and confirmed that samples were collected at sites with different levels of contamination.

### Community composition differs between river and hospital waters

Metagenomic DNA isolated from the water samples was used to determine community taxonomic profiles. Although both RS samples yielded low amounts of DNA (<1 ng/µl), in agreement with the low bacterial recoveries upon plating, it was impossible to PCR-amplify the 16S rRNA gene from the sample collected in 2015 (RS15) and it was therefore not further used. Amplicon libraries done with the remaining 11 samples were sequenced and, after quality filtering and merging, the number of clean reads ranged from 41,207 to 65,614 per sample. Diversity analysis of these samples was performed by randomly subsampling each dataset to 41,000 sequences. The estimated richness by direct OTU count (observed OTUs) was highest for RL15, but after being corrected by phylogenetic relatedness (Faith’s PD) it was highest for the upstream river samples (source and middle section), followed by the lower section (RL) waters and the hospitals (Kruskal-Wallis tests: observed OTUs, *p* = 0.03762; Faith’s PD, *p* = 0.01672) (Table 3). However, Shannon’s diversity index, which considers taxa abundances, did not show significant differences among upstream (RS and RM), downstream (RL) and hospital waters (Kruskal-Wallis test, *p* = 0.8275).

**Table 3:** Alpha diversity measures for microbial composition and ARGs.

Sample^a^	Taxa			ARGs	
	Observed OTUs	Phylogenetic Diversity	Shannon	Shannon	Simpson
RS16	831	60.10	4.99	4.75	0.96
RM15	779	59.56	6.07	4.02	0.92
RM16	849	61.26	5.56	3.75	0.92
RL15	877	56.71	7.03	5.44	0.97
RL16	750	52.41	4.98	5.65	0.97
HA15	718	45.54	6.37	6.32	0.98
HA16	708	46.62	5.30	5.25	0.97
HB15	565	37.89	5.93	5.19	0.96
HB16	524	37.36	4.55	6.40	0.98
HC15	752	46.01	6.35	6.77	0.99
HC16	690	45.51	6.15	6.58	0.98

^a^Sample names as described in Table 1.

**Figure 2:**
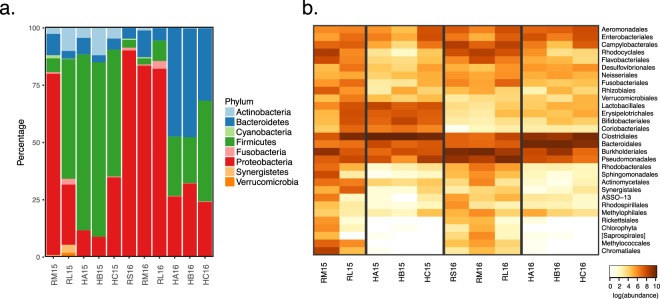
Abundance of bacterial taxa based on 16S rRNA gene analysis. (**a**) Abundance of bacterial phyla; (**b**) Heat map of the abundance of bacterial orders. RS = river source; RM = middle section; RL = lower section; HA, HB, HC = hospitals A, B and C; 15 = 2015; 16 = 2016.

**Figure 3:**
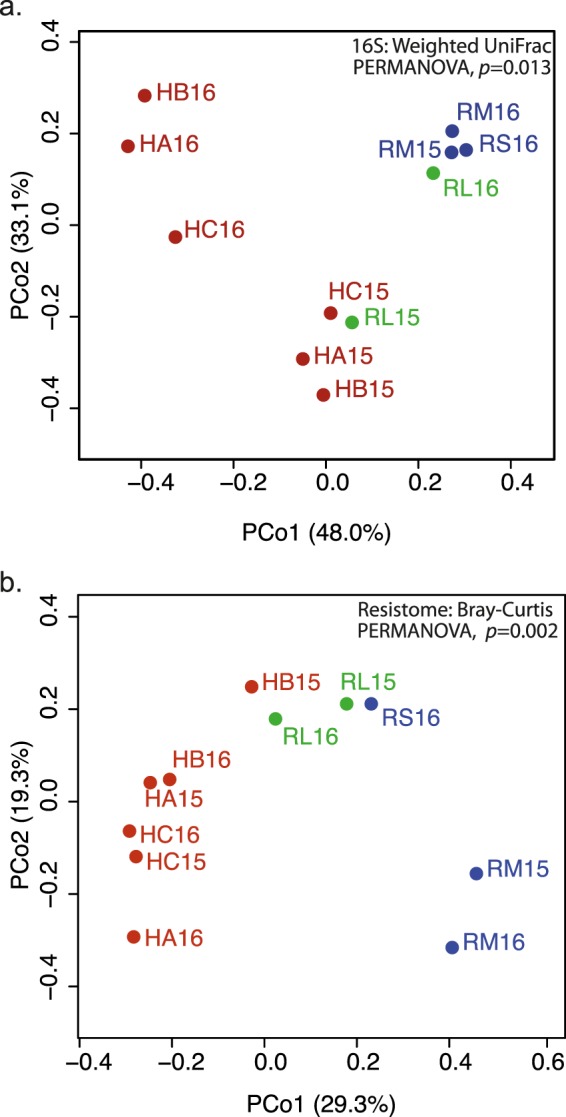
Principal coordinates analyses of taxonomic and ARG diversity. PCoAs were done with (**a**) weighted UniFrac distances of OTU composition and (**b**) Bray-Curtis distances of ARG profiles. Sample names as described in Fig. 2.

Taxonomic assignment was done at various levels using the naïve Bayesian RDP Classifier^28^. A phylum-level examination (Fig. 2a) indicated that hospital and river samples differed from each other, except for the lower river sample collected in 2015 (RL15), which had a profile similar to hospital samples. This pattern was also observed when considering the other taxonomic levels. At the order level, hospitals and the RL15 sample had a very low abundance or complete absence of the Rickettsiales, in contrast to what was observed in the other river samples (>100-fold difference) (Fig. 2b). The time of sampling also affected community composition, as seen for example by the predominance of the phylum Bacteroidetes in hospital samples from 2016 and Actinobacteria in 2015 samples. A linear discriminant analysis (LDA) was done to identify differentially enriched taxa. The RL samples were excluded and analyzed in detail below, since they represent a sink that combines water from both upstream river sites and urban discharges, including the hospitals wastewaters. The LDA confirmed enrichment of the Firmicutes phylum in hospitals, and the phyla Proteobacteria, Fusobacteria and Cyanobacteria in upstream water samples (RS and RM) (Supplementary Fig. S1, Supplementary Table S2). Genera associated with environmental microbial communities, like *Methylosinus* (methanotroph), *Thiocapsa* (sulfur-reducing), *Rhodobacter* (photosynthetic), *Limnohabitans* (planktonic), *Dechloromonas* (aromatic-compound-oxidizing) and *Polynucleobacter* (planktonic), were enriched in the upstream samples; while genera associated with the human gut microbiota, such as *Faecalibacterium*, *Roseburia*, *Bacteroides* and *Ruminococcus* were more abundant in hospitals (Supplementary Fig. S1, Table S3). Finally, a principal coordinates analysis (PCoA) done with weighted UniFrac distances explained 81.1% of the total variation within the first two principal coordinates and showed three clusters: hospital samples from 2016, RL15 and hospital samples from 2015, and RL16 with the upstream river samples (Fig. 3a). The first component, which explains 48.0% of the variation, mainly separates hospitals from river samples. These analyses revealed the existence of community differences between hospital, upstream and downstream river waters (PERMANOVA, *p*≈0.013), and showed that the two samples from the river’s lower section were different from each other, one featuring more taxa enriched in hospitals and the other more taxa enriched in upstream river locations.

Whole metagenomic libraries were also constructed and sequenced in an Illumina NextSeq. Again, the RS15 sample was excluded because library construction failed. After processing and filtering for quality, sequence reads ranged from 1,050,016 to 14,108,499 reads per sample. These metagenomic reads were used to assess microbial community composition with Metaxa2^29^. The broad taxonomic patterns obtained were consistent with those observed by doing 16S rRNA gene profiles, though Metaxa2 failed to identify phyla of low abundance (Supplementary Fig. S2), as expected for a shotgun analysis.

### ARG abundance and diversity are greater in more intervened locations

**Figure 4:**
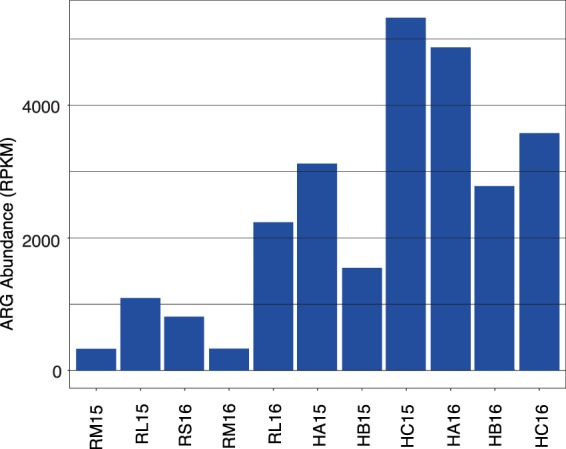
ARG counts in the water samples. Sample names as described in Fig. 2.

ShortBRED^30^ was used along with 2,937 ARG markers generated using the CARD^31^ and Lahey Clinic β-lactamase databases to quantify ARGs in the metagenomic samples. Counts were normalized to gene length and sequencing depth per sample (RPKM)^32^, and to the bacterial proportion of 16S copies found by Metaxa2 (Supplementary Table S4). This analysis showed a higher abundance of ARGs in hospital samples, followed by lower section (RL) and upstream river waters (RS and RM) (Fig. 4). In addition, the RL16 sample had an abundance of ARGs comparable with the hospitals and, intriguingly, more ARGs were found in RS than in RM. These RPKM counts show that, in general, waters with a greater level of anthropogenic influence harbor more ARGs. We observed the same tendency when considering only clinically relevant ARGs and genetic determinants^33,34^ (Supplementary Fig. S3). These results indicate that markers present in pathogens were found in water samples, even in upstream waters considered to be less impacted by human activities. However, given that their identification relies on bioinformatic searches against a known database, in this case the curated CARD database that focuses on known resistance genes, novel unknown markers will be missed. The large number of ARGs detected by sequencing contrast with the small number of recovered resistant microorganisms and underscore the discrepancy between methods to assess natural communities where the vast majority of microbes are non-culturable in laboratory media.

**Figure 5:**
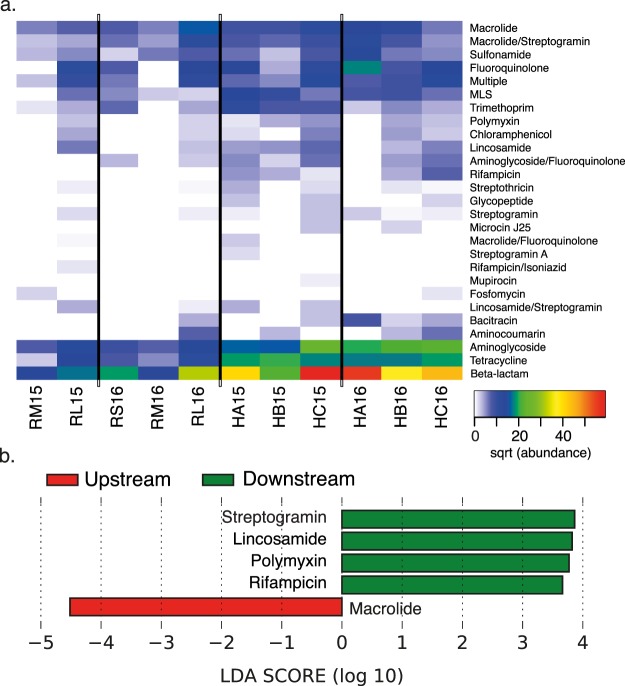
Detection of ARGs in water samples. (**a**) Abundance of ARGs classified by target antibiotic; (**b**) Differentially enriched resistances in hospitals and upstream river waters. Sample names as described in Fig. 2.

In addition to having greater ARG counts, hospitals and the river’s lower section also had higher ARG diversity (Kruskal-Wallis tests: Shannon’s index, *p* = 0.0441; Simpson’s index, *p* = 0.0307) (Table 3). This is illustrated, for example, by resistances to polymyxin and chloramphenicol, which were present in hospitals and RL samples but not in the upstream river samples (Fig. 5a). An LDA indicated that hospitals were enriched with resistance to streptogramins, lincosamides, polymyxins and rifampicin, while upstream samples were only enriched with resistance to macrolides (Fig. 5B, Supplementary Table S5). Furthermore, 28 ARGs were more abundant in hospitals, compared to only five in upstream samples (Supplementary Fig. S1, Supplementary Table S6). ARGs within a given antibiotic category, such as macrolide resistance, also differed between upstream river samples and the hospital and RL samples (Supplementary Fig. S4). A principal coordinates analysis done using Bray-Curtis distances (48.6% variation explained in the first two components) coincided with this observation and showed that hospital and RL samples clustered together and were apart from the other river samples (Fig. 3b). These results show that the diversity of ARGs is greater in more contaminated hospital and RL waters and that these markers differ with respect to upstream river samples (PERMANOVA, *p* = 0.002).

### Bacterial taxa and ARGs in RL samples can be traced to hospitals or upstream waters

**Figure 6:**
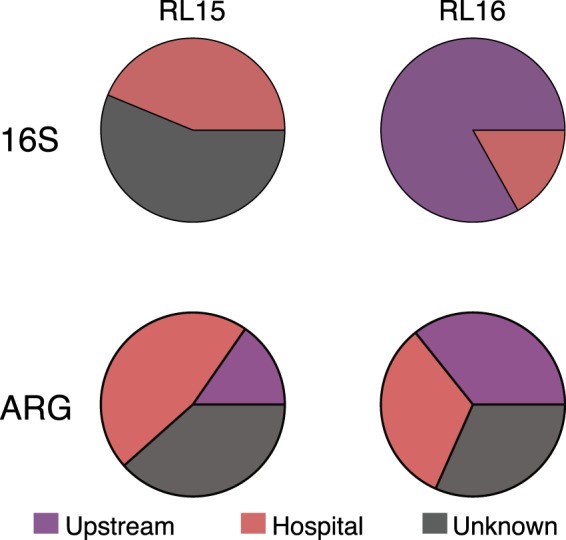
Source tracking in the river lower section (RL) samples. Origin of genera (16S) and ARGs, as traced to hospitals or upstream waters using SourceTracker.

By running a Procrustes analysis we found that the association among samples, based on taxonomic composition, correlated with the association given by resistome (Procrustes, *p* = 0.008, *M*^2^ = 0.554), suggesting that some of the resistances could be associated with certain taxa observed at specific locations. Given that the river’s lower section (RL) is influenced by both upstream waters and urban effluents, including hospital wastewaters, SourceTracker^35^ was used to identify the potential source of both microbial taxa and ARGs present in the downstream river samples (RL). In the case of taxa, the source of the bacterial genera detected in the RL samples from different years, RL15 and RL16, had different origins. RL15 genera originated solely from unknown sources (56.2%, *SD* = 2.6%) and hospitals (43.8%, *SD* = 2.6%), while the composition of RL16 was derived mainly from the upstream RS and RM sources (83.1%, *SD* = 1.5%) (Fig. 6). The resistomes in RL samples also had different origins: in RL15, most ARGs originated from hospital wastewaters (46.2%, *SD* = 2.5%), followed by unknown sources (38.4%, *SD* = 2.0%) and upstream river sources (15.4%, *SD* = 1.3%); while in RL16, most genes could be traced to upstream river sources (35.7%, *SD* = 1.3%), followed by hospital wastewaters (32.6%, *SD* = 2.8%) and unknown sources (31.6%, *SD* = 1.9%). The significant contributions from unknown sources were expected because contaminants released into the river have numerous additional origins, like agricultural activities and household discharges. When considering only the known sources, again RL15 had a larger ARG contribution from hospitals and RL16 from upstream river locations (RS and RM) (Fig. 6). The lower river is therefore a site that brings together microbial communities and resistomes from diverse sources, including upstream and hospital wastewaters.

### Risk of horizontal gene transfer

To evaluate possible associations between ARGs and mobile genetic elements that could facilitate horizontal gene transfer, we assembled contigs using GenSeed-HMM^36^ and SPAdes^37^ and then annotated them using blastx^38^. The GenSeed-HMM assembly used HMMs derived from common mobilization genes and antibiotic resistance to seed the assembly, thus only generating contigs that contained either antibiotic resistance genes or mobilization genes (or both). This strategy generated a total of 57 contigs, for all samples, 10 with a size larger than 1,000 bp. In four of these contigs we found co-occurrence of these markers: two cases in sample HC15 (Sul1 and an integrase family protein such as IntI1, and AadA and an integrase family protein), and single occurrences for HC16 (Sul1 protein and an integrase family protein) and RL15 (streptomycin adenylyltransferase gene). The SPAdes global assembly was also searched for contigs with co-occurrence of ARGs and mobile genetic elements. In this case, we identified four such contigs: in samples HB16 (AAC(6′)-Ib gene with IntI1 integrase), HC16 (*qacH* gene with IS6100 transposase), RL16 (AAC(6′)-Ib gene with IntI1 integrase) and RM15 (*mphD* gene with IS26 transposase). Of these 8 contigs with evidence of co-localization for both an ARG and a mobilization gene, only one was from the river middle section and none were from the river source, suggesting a higher potential for horizontal gene transfer in densely populated communities as those of the hospitals and the lower section of the river.

## Discussion

In this work, we characterized aquatic microbial community composition and the resistome in water samples taken at sites with varying degrees of contamination: three locations along the Bogotá River and the wastewaters from three hospitals in the city of Bogotá. Physicochemical measures, bacterial counts and antibiotic and pharmaceutical quantifications indicated that human activities had a negative impact on water quality, consistent with previous reports^25^. The presence of drugs of common clinical use in these water samples reflects both drug usage^26^ and contamination^1,39^. Antibiotics were present in low amounts that, even if not effective for selection of resistant bacteria^33^, particularly in the upstream river samples, may nonetheless alter microbial signaling and affect interactions^40^. It is important to keep in mind that, given the limited sampling scheme and the variable drug concentrations obtained, these measurements are not meant as a comprehensive analysis and are thus difficult to correlate with microbial populations. Importantly, however, the detection of these drugs provides evidence that they were present, even though their concentration and effects at other locations or microenvironments, such as biofilms, are unknown. Concentrations in hospital effluents showed variability, possibly due to usage and stability, and were similar to those found in other hospital wastewaters from Colombia^41^. These locations may serve as sites for gene exchange and selection of resistant microorganisms^20^, even at sub-inhibitory concentrations^17^.

The changes observed in water quality were accompanied by alterations in microbial community composition and ARG profiles. The taxa differentially enriched in the upstream (RS and RM) and hospital samples were indicative of different ecological contexts. Environmental microbes, including methanotrophic, sulfur-reducing, photosynthetic and aromatic-compound-oxidizing bacteria, were enriched in the upstream samples, while taxa associated with the human gut microbiota were more abundant in hospitals, consistent with human fecal contamination. Both PCoA and source tracking analyses showed that communities from downstream samples (RL) could be derived from upstream river and hospital wastewater sources. For example, the abundance of Firmicutes in the RL15 sample could be explained by the presence of this discriminant taxon in hospital samples (Fig. 2a, Fig. 6). However, this finding is surprising given that hospital discharges into the river should account for a minimal percentage of the total effluents, suggesting that the fecal contamination of the lower section of the river is extremely high. Intriguingly, the two RL samples showed noticeable differences: the 2015 sample was more taxonomically similar to hospital samples and the 2016 sample to upstream river samples. This difference was marked by particular taxa, such as the increased abundance of Clostridiales in RL15. This disparity, which could be due to variations in experimental procedures, such as DNA extraction methods, may also be due to factors such as: (i) environmental conditions or raining patterns, like the lower-than-average precipitation registered for the 2015 sampling period^42^; or (ii) rural and urban discharges into the river at the different sampling times. Additional sampling would be required to define possible causation and specific correlations with environmental variables.

The resistome varied both in abundance and diversity. ARG abundance followed a gradient that correlated with microbial community shifts and was greater in the downstream sites than in the upstream river samples, with a marked increase in hospital samples that also had greater presence of antibiotics. The higher ARG abundance and diversity in the river source sample RS16, when compared to the river’s middle section, confirmed the natural occurrence of these markers in the environment^6,43^. The drop in abundance of these genes in the river mid-section (RM samples) in both years was intriguing and could be due to multiple urban, agricultural and industrial discharges^25^ that change microbial communities and dilute the microbial resistances. This location can also constitute a transition state from a non-contaminated to a more contaminated environment. More closely spaced samplings could be done to address this issue. The intermediate abundance of ARGs in downstream river samples (RL) is consistent with this location being a sink that combines water and communities from upstream river and urban sources. Clinically relevant ARGs were also detected in all sites studied and follow the overall trend seen for ARGs in general, with greater abundance in hospital and more contaminated water samples. On the other hand, many environmental ARGs could have been missed given our limited knowledge of environmental ARG diversity and ability to detect them, and the bias of public databases towards the most frequently encountered genes. These issues highlight the importance of thoroughly curating databases and doing functional evaluation of resistant phenotypes. In this study, the limited amount of DNA recovered made it impossible to carry out functional metagenomics to detect expressed resistance markers^44^. Future studies would require collecting larger amounts of water per site and/or improving extraction protocols in order to obtain sufficient high-quality DNA.

The similarity of ARG profiles in contaminated sites (RL and hospital samples) and their differentiation from upstream river locations are also indicative of composition being constrained by ecology^45^, as hospital effluents released to the environment impact and alter microbial communities. The RL16 sample, which clustered close to upstream river samples when based on taxonomy and to hospital samples when based on resistances, can also reflect differences in terms of the relative contributions of microbes and their resistance genes in the incoming contaminating effluents and how these affect the resulting community. Given the low-depth Illumina sequencing and the limited number of contigs obtained, the co-occurrence of mobilization genes and ARGs in contigs mainly from hospital and RL samples suggests potential for horizontal gene transfer. Isolation of microorganisms or deeper sequencing would be needed to identify specific markers, mobile elements and genetic backgrounds that could help to accurately trace the genes and assess the risks posed by these ARGs^33,46^.

Despite the limited sampling scheme, this study shows that ARGs and pathogens are present in this freshwater ecosystem and are influenced by anthropogenic contamination, an important driver of micro-scale environmental changes. This influence is demonstrated by the presence of antibiotics, drugs and high-risk ARGs^46^ in downstream river samples and by the similarity between these and hospital samples. The identification of known clinical pathogens such as methicillin-resistant *Staphylococcus aureus* (MRSA), vancomycin-resistant enterococci (VRE) and resistant enterobacteria also exposes the risk associated with use of this water supply for diverse activities such as animal farming and crop irrigation^47^ that can promote dissemination of undesired microorganisms and their genes into the environment and the food chain^11,12,48^. The close proximity between genes associated with mobile genetic elements and ARGs, especially those considered to be of high risk to human health such as *sul1* and AAC(6′)-Ib^33^, raises the possibility that these genes may be mobilized among microorganisms in these new settings. These results emphasize the need for surveillance of microbiological risks in this river ecosystem, which can be done with metagenomics^49,50^, and implementation of effective strategies for water management aimed at avoiding undesired spread of microorganisms and resistance determinants due to the cumulative effect of small-scale contaminating discharges^39^.

This study provides a snapshot and brief survey of microbial communities and their resistance genes at two points in time and at geographically close sites along the Bogotá River. Sampling at additional locations, periodic sampling at the same sites, as well as greater sample volumes and depth of sequencing, would provide a broader picture of the dynamics of the microbial communities and their resistomes and allow correlation with particular environmental variables. However, our metagenomic data shows that microbial communities and the resistomes of this river ecosystem are heavily impacted by anthropogenic activities and change as a consequence of contamination. These results also illustrate how human contamination can drive the spread of ARGs in this river ecosystem and generate conditions for microbial communities and antibiotic resistance genes of diverse origins to come together, mix and disseminate, posing risk to the local population. Further work is needed, however, to understand the risk of dissemination of these determinants from contaminated sites, such as hospitals, into environmental water supplies^39^.

## Methods

### Sampling sites and DNA extraction

Water samples were taken in 2015 (between August and November) and 2016 (between May and June), both during the rainy season, but with slightly higher rainfall in 2016^42^. Samples were obtained from three different locations along the Bogotá River (three samples of 18L per site, collected on three different days in 2015, and one sample of 18L per site in 2016), and three hospitals (three raw wastewater samples of 6L on different days per site in 2015, and one sample of 6L per site in 2016) in the city of Bogotá, Colombia (Fig. 1). The hospital wastewaters are monitored to comply with regulatory standards regarding microbial counts. Smaller volumes were collected from hospital effluents since these had higher biomass and were sufficient to obtain total community DNA. The differences in total sampled volumes between 2015 and 2016 were due to site accessibility and permits. The river samples were collected at three locations with distinct levels of anthropogenic intervention: the source (RS), which is in the mountains and has little human intervention (Villapinzón, Cundinamarca; 5° 15′ 51.82″ N; 73° 32′ 26.72″ W); the middle section (RM), just before the river enters the city of Bogotá but after it receives water effluents from small urban areas and local industries (Cota, Cundinamarca; 4° 47′ 57.0″ N; 74° 05′ 45.4″ W); and the lower section (RL), right after the city of Bogotá, where the river receives wastewater discharges (Soacha, Cundinamarca; 4° 34′ 41.6″ N; 74° 15′ 08.2″ W). Hospital names have been kept anonymous and their locations were verified to be connected to the city’s sewage system, which ultimately discharges into the Bogotá River in locations between the second (RM) and third (RL) sampling site (URL: mapas.bogota.gov.co). Samples were transported on ice to the lab and processed the same day they were collected by pre-filtering (30 µm Whatman®) to eliminate large particles and then filtering through 0.45 µm mixed cellulose ester filters (PALL) and 0.2 µm cellulose acetate filters (Sartorius) using a vacuum filtration system (PALL). Biomass from 0.45 and 0.22 µm filters was recovered by placing filters in 50 mL tubes with PBS, 0.05% Tween 20 (Sigma-Aldrich) for 30 min at 28 °C with agitation, followed by vortexing three times and then removing filters and centrifuging at 4,696 × g for 30 min at 4 °C. DNA was obtained from the recovered biomass with two methods. For the 2015 samples, biomass was eluted using 2.5 mg/mL lysozyme (Sigma-Aldrich) for 1 h at 37 °C, adding 200 µg/mL proteinase K (Invitrogen) for 3 h at 56 °C, then extracting with phenol:chloroform:isoamylic alcohol (25:24:1) and precipitating with 0.6 volumes of isopropanol and 0.3M sodium acetate by centrifugation at 16,200 × g for 30 min. For the 2016 samples, DNA was obtained using the DNeasy Kit (Qiagen). All DNA samples were quantified using the Qubit dsDNA HS Assay (ThermoFisher Scientific) and stored at −80 °C. For analysis of antibiotics and other pharmaceuticals, the samples stored at −80 °C (approx. 50 mL) were shipped in cool containers to the University Jaume I, Spain.

### Water analysis and determination of pharmaceuticals

Physicochemical analyses (chemical oxygen demand (COD), biological oxygen demand (BOD), total nitrogen (TN) and total suspended solids (TSS)) were performed on the 2015 samples at Biopolab Ltda. (Bogotá, D. C., Colombia), following standard protocols^51^.

The samples were stored at −18 °C until they were analyzed for the presence of the antibiotics metronidazole, sulfamethoxazole, trimethoprim, norfloxacin, ciprofloxacin, clindamycin, clarithromycin, erythromycin and azithromycin; and the drugs carbamazepine, venlafaxine, losartan, irbesartan, valsartan, naproxen and diclofenac. On the day of the analysis, samples were thawed, centrifuged and an aliquot was used for direct analysis by ultra-high-performance liquid chromatography coupled to tandem mass spectrometry with triple quadrupole analyzer (UHPLC-MS/MS), using electrospray ionization^52^ (Supplementary Methods).

### Culture conditions

Water samples (400 mL) were serially diluted in sterile distilled water and filtered through 0.45 µm cellulose nitrate filters (Sartorius) and GN-6 Metricel MCE Membrane Disc Filters (Pall), which were placed on Chromocult® Coliform Agar (Merck Millipore) for 24 h at 37 °C for detection of *E. coli* (blue colonies), total coliforms (salmon-colored colonies) and other Gram-negative bacteria (transparent colonies). Resistant bacteria were recovered by plating on chromogenic media selective for β-lactamase producing Enterobacteriaceae (CARBA Agar, ChromID, BioMerieux) and extended-spectrum β-lactamases (ESBL Agar, ChromID, BioMerieux), methicillin-resistant *S. aureus* (MRSA Agar, ChromID, BioMerieux), and vancomycin-resistant enterococci (VRE Agar, ChromID, BioMerieux). Recovered colonies were subjected to previously reported PCR assays for species identification and resistance detection^53–60^.

### 16S rRNA gene amplification, sequencing and processing

The V4 region of the 16S rRNA gene was amplified using primers 515F and 806R that include adaptors for Illumina flow cells (http://www.earthmicrobiome.org/protocols-and-standards/16s/)^61^. PCR amplifications were done by triplicate in a 96-well plate in a 25 µL volume containing 10.2 µL H_2_O, 12.8 µL Takara Premix Taq DNA Polymerase (TaKaRa, #R004A), 1 µL 515F forward primer (10 µM), 0.5 µL 806R reverse primer (10 µM) with the unique identifier and 0.5 µL DNA (1 ng/µL). PCR reactions were done as follows: 98 °C for 30 s, 35 cycles of 98 °C for 10 s, 50 °C for 30 s and 72 °C for 30 s, and a final extension at 72 °C for 120 s. PCR products were purified using the Agencourt AMPure XP PCR Purification kit (Beckman Coulter, A63880), quantified using the Picogreen kit (Invitrogen, P11496), and loaded with a concentration of 8 pM with 25% PhiX spike-in to an Illumina MiSeq platform, where they were sequenced using 250 bp paired ends^62^.

Sequence data was de-multiplexed by sample with the Qiime 1^63^ script split_libraries_fastq.py (-p 0.1 -n 500) and operational taxonomic units (OTUs) were generated using UPARSE^62,64^ (usearch8.1), which included merging of forward and reverse reads (-fastq_mergepairs, -fastq_maxdiffs 0 -fastq_truncqual 3 -fastq_maxmergelen 258 -fastq_minmergelen 248), quality filtration (-fastq_filter, -fastq_maxee 0.5), de-replication (-derep_fulllength) and sorting (-sortbysize, -minsize 2), clustering (-cluster_otus), checking for chimeric sequences against the Gold database (using the -uchime_ref and -db flags; database downloaded from http://drive5.com/uchime/uchime_download.html), mapping back of reads to OTUs at 97% identity (using the -usearch_global and -db flags, and setting -strand to both); and generation of the final OTU table using the script uc2otutab.py^65^. Taxonomy was assigned using the RDP Classifier^28^ against the Greengenes database^66^ (with Qiime 1 scripts align_seqs.py, assign_taxonomy.py, filter_alignment.py and make_phylogeny.py).

### Metagenomic sequencing and processing

DNA samples were processed using the Nextera DNA Library Prep Kit (Illumina) with modifications^67^, and libraries were sequenced using an Illumina NextSeq (Center for Genome Sciences and Systems Biology, Washington University School of Medicine). Reads were de-multiplexed by the sequencing center, analyzed for quality using FastQC (Babraham Bioinformatics), and quality filtered with Trimmomatic^68^ (-phred 33, HEADCROP: 10, ILLUMINACLIP: NexteraPE, LEADING: 3, TRAILING: 3, SLIDINGWINDOW: 4:20, MINLEN: 36). Filtered reads were used for microbial community and antibiotic resistance gene analyses. Metaxa2^29^ was used to assess microbial community composition from metagenomic reads^62,69^.

### Survey of antibiotic resistance genes in the metagenomic reads

ShortBRED was used to determine the presence and the abundance of ARGs in metagenomic reads^30,70^. ShortBRED markers were generated based on the Comprehensive Antibiotic Resistance Database (CARD) (downloaded 6 March 2017; 2,158 proteins)^31^ and the Lahey Clinic β-lactamase database provided by Erica Pehrsson (1,146 proteins; only the short VEB-6 protein was removed), clustered at 100% identity^70^. As a reference database to remove constitutive bacterial genes, we used the UniRef90 protein dataset, as suggested^30^ (52,629,880 entries). ShortBRED generated 2,937 markers that it used to quantify ARGs in each water sample, producing files containing each marker family and its abundance, normalized to reads per gene kilobase per million reads (RPKM). These marker families were classified according to target using the hand-curated metadata file of the CARD and Lahey Clinic β-lactamase databases, provided by Erica Pehrsson (Supplementary Table S7)^70^.

### Alpha and beta diversity analyses

Alpha and beta diversity analyses were carried out under the Python 2.7 environment for both community composition and ARG profiles. OTU tables were rarefied to 41,000 sequences per sample using Qiime 1^63^. Observed OTUs, Shannon’s index and Faith’s phylogenetic diversity, which incorporates phylogenetic differences between species, were calculated using the script alpha_diversity.py. ARG tables were not rarefied since RPKM abundances need no further normalization in shotgun sequencing, and only Shannon’s and Simpson’s indexes in each sample were determined. Richness and diversity indexes were compared between upstream (RS and RM, n_1_ = 3), downstream (RL, n_2_ = 2) and hospital (n_3_ = 6) samples with Kruskal-Wallis tests (α = 0.05). For beta diversity analyses, the community composition of each sample was compared using a weighted UniFrac distance matrix calculated with the Qiime script beta_diversity.py, and the same script was used with the ARG table to calculate a Bray-Curtis distance matrix. PCoA plots were generated from the distance matrices using the script principal_coordinates.py. Permutational analysis of variance (PERMANOVA) was carried out independently with both distance matrices to compare upstream, downstream and hospital samples using the script compare_categories.py and 999 permutations (α = 0.05), while using the year of sampling as a covariable. A Bray-Curtis distance matrix was also calculated for community composition and was used along the resistome matrix for a Procrustes analysis using the script transform_coordinate_matrices.py and 999 permutations (α = 0.05).

### Identification of discriminatory features

Given our small sample size and the lack of a normal distribution for the data, we chose LEfSe^70,71^, which performs non-parametrical statistical tests, to determine differentially enriched OTUs and ARGs in upstream and hospital samples. OTU tables for phyla and genera were used as inputs for biomarker taxa discovery in LEfSe. The ARG table was also used as input for LEfSe after removing undetected ARGs and classifying the remaining according to the type of antibiotic to which they confer resistance. For both OTU and ARG comparisons, alpha was set at 0.05 for the Kruskal-Wallis and the pairwise Wilcoxon rank-sum tests. No multiple-testing correction is necessary as significant results are reported only if all the pairwise Wilcoxon rank-sum tests are significant. The linear discriminant analysis effect size threshold was set at 3.0 and the program performed all-against-all comparisons.

### Tracking of OTUs and ARGs

SourceTracker^35^ was used to determine the proportion of OTUs and ARGs of each sample from the river’s lower section that were associated predominantly with either hospitals or the river upstream of the city (river source and middle section).

### Analysis of mobile genetic elements

GenSeed-HMM^36^ was used to assemble the metagenomic reads using hidden Markov models (HMMs) of integrase genes and high-risk ARGs. Fifty-eight integrase HMMs were downloaded from the Gypsy Database 2.0 (http://gydb.org/index.php/Collection_HMM)^72^ and 37 HMMs were generated using HMMER 3.1^73^, after alignment of high-risk indicator proteins and their closest sequences obtained from the NCBI. These relevant proteins were obtained from the risk classification done by Martínez, Coque & Baquero^46^, from the risk indicator ARGs outlined by Berendonk *et al*.^33^ and from the contigs assembled by Forsberg *et al*.^74^. All HMMs were used as seeds for the GenSeed-HMM assembly and contigs were searched for ARGs and mobile genetic elements using blastx^38^ against the nr database (E value threshold: 0.001, percent identity threshold: 90%).

The metagenomic reads were assembled into contigs with SPAdes^37^. Blastx (E value threshold: 0.001; percent identity threshold: 90%) was used to search for ARGs and mobile genetic elements using a custom database containing all entries from the ARG database generated in this study and the ACLAME database of mobile genetic elements (downloaded 12 August 2017)^75^.

## Supplementary information

Supplementary Material

Table S7

## Data Availability

Sequence data of both the bacterial 16 S rRNA genes and the whole metagenomes were deposited in the European Nucleotide Archive under project accession number PRJEB22915 (http://www.ebi.ac.uk/ena/data/view/PRJEB22915).
