# Supplementary material for: Bogotá River anthropogenic contamination alters microbial communities and promotes spread of antibiotic resistance genes
Source: Sci Rep. 2019 Aug 13;9:11764. doi: 10.1038/s41598-019-48200-6 (PMC6692338; doi:10.1038/s41598-019-48200-6)
Supplement: Supplementary file 1 — Supplementary Material [file 41598_2019_48200_MOESM1_ESM.pdf]

Bogotá River anthropogenic contamination alters microbial communities and promotes spread of antibiotic resistance genes

Carlos Eduardo Posada-Perlaza[a,b,c], Adán Ramírez-Rojas[c], Paola Porras[d], Boahemaa Adu-Oppong[e], Ana-María Botero-Coy[f], Félix Hernández[f], Juan M. Anzola[c], Lorena Díaz[d], Gautam Dantas[e,g,h,i], Alejandro Reyes[a,b,e,#], María Mercedes Zambrano[c,#]

a) Computational Biology and Microbial Ecology Group, Department of Biological Sciences, Universidad de los Andes, Bogotá, D. C., Colombia; b) Max Planck Tandem Group in Computational Biology, Universidad de los Andes, Bogotá, D. C., Colombia; c) Molecular Genetics and Bioinformatics, Corporación CorpoGen, Bogotá, D. C., Colombia; d) Molecular Genetics and Antimicrobial Resistance Unit, International Center for Microbial Genomics, Universidad El Bosque, Bogotá, D. C., Colombia; e) Center for Genome Science and Systems Biology, Washington University School of Medicine, St. Louis, MO, USA; f) Research Institute for Pesticides and Water, University Jaume I, Castellón, Spain; g) Department of Pathology and Immunology, Washington University School of Medicine, St. Louis, MO, USA.; h) Department of Molecular Microbiology, Washington University School of Medicine, St. Louis, MO, USA; i) Department of Biomedical Engineering, Washington University, St. Louis, MO, USA.

#Corresponding authors: María M. Zambrano, mzambrano@corpogen.org, Corporación Corpogen, Carrera 5 #66A-34, Bogotá, Colombia; Alejandro Reyes, a.reyes@uniandes.edu.co, Universidad de los Andes, Carrera 1 #18A-70, Bogotá, Colombia.

Supplementary Methods

Determination of pharmaceuticals

Duplicate samples were analyzed by UHPLC-MS/MS, using electrospray ionization, for the presence of antibiotics and other pharmaceutical compounds. Analysis was made by direct injection of the sample, adding the corresponding isotope-labeled internal standard (ILIS), when available, for matrix effects correction. In total, six ILIS were applied for antibiotics (sulfamethoxazole, trimethoprim, norfloxacin, ciprofloxacin, erythromycin, azithromycin) and five ILIS for other drugs (carbamazepine, venlafaxine, irbesartan, valsartan, diclofenac). Up to three MS/MS transitions were acquired, using the most sensitive for quantification (Q) and the other two for confirmation of the identity of the compound detected (q1, q2). The agreement in retention time and the compliance of at least one ion intensity ratio (q1/Q or q2/Q) between sample and reference standard was required for a reliable identification. Quality control samples (QCs) were prepared and analyzed together with the samples in every sample sequence to support the methodology applied. QCs consisted of samples from the Bogotá River's lower section (RL), which were fortified with the analytes under study at 0.1, 1.0 and 10 μg/L. A recovery range between 60 and 140% was considered as acceptable, as established in some guidelines for residue analysis (European Commision Directorate-General for Health and Food Safety, 2015). QCs showed that, in most cases, recoveries could not be estimated at the lowest level tested (0.1 μg/L). Therefore, medium or high fortification level (1.0 and 10 μg/L) recoveries were used. These were in the range 70-122% with the only exception of clarithromycin, which presented a mean recovery of 147%, surely due to the presence of this compound at concentrations above 1 μg/L in most of the samples (**Table 1**); this made the calculation of

recovery, by subtracting the concentration of the "blank" sample, troublesome. Although lower concentrations could be quantified for some compounds, the lowest level reported in this work was 0.01 μg/L, in order to reduce uncertainty in the quantification and in the identification process in this type of complex-matrix samples.

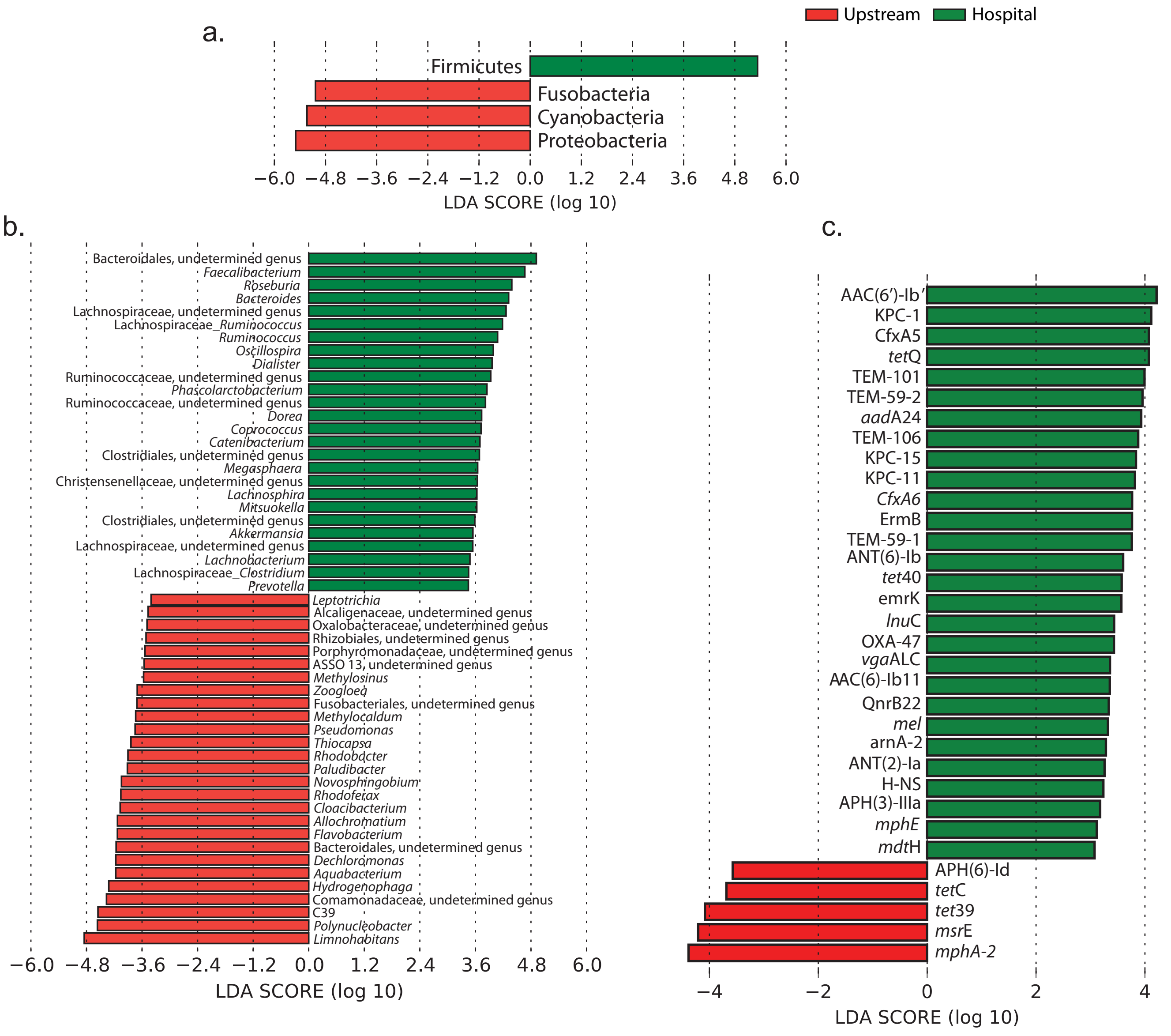

Upstream
Hospital
a.
Firmicutes
Fusobacteria
Cyanobacteria
Proteobacteria
−6.0 −4.8 −3.6 −2.4 −1.2 0.0 1.2 2.4 3.6 4.8 6.0
LDA SCORE (log 10)
b.
Bacteroidales, undetermined genus
Faecalibacterium
Roseburia
Bacteroides
Lachnospiraceae, undetermined genus
Lachnospiraceae_Ruminococcus
Ruminococcus
Oscillospira
Dialister
Ruminococcaceae, undetermined genus
Phascolarctobacterium
Ruminococcaceae, undetermined genus
Dorea
Coprococcus
Catenibacterium
Clostridiales, undetermined genus
Megasphaera
Christensenellaceae, undetermined genus
Lachnosphira
Mitsuokella
Clostridiales, undetermined genus
Akkermansia
Lachnospiraceae, undetermined genus
Lachnobacterium
Lachnospiraceae_Clostridium
Prevotella
Leptotrichia
Alcaligenaceae, undetermined genus
Oxalobacteraceae, undetermined genus
Rhizobiales, undetermined genus
Porphyromonadaceae, undetermined genus
ASSO 13, undetermined genus
Methylosinus
Zoogloea
Fusobacteriales, undetermined genus
Methylocaldum
Pseudomonas
Thiocapsa
Rhodobacter
Paludibacter
Novosphingobium
Rhodoferax
Cloacibacterium
Allochromatium
Flavobacterium
Bacteroidales, undetermined genus
Dechloromonas
Aquabacterium
Hydrogenophaga
Comamonadaceae, undetermined genus
C39
Polynucleobacter
Limnohabitans
−6.0 −4.8 −3.6 −2.4 −1.2 0.0 1.2 2.4 3.6 4.8 6.0
LDA SCORE (log 10)
c.
AAC(6′)-Ib′
KPC-1
CfxA5
tetQ
TEM-101
TEM-59-2
aadA24
TEM-106
KPC-15
KPC-11
CfxA6
ErmB
TEM-59-1
ANT(6)-Ib
tet40
emrK
lnuC
OXA-47
vgaALC
AAC(6)-Ib11
QnrB22
mel
arnA-2
ANT(2)-Ia
H-NS
APH(3)-IIIa
mphE
mdtH
APH(6)-Id
tetC
tet39
msrE
mphA-2
−4 −2 0 2 4
LDA SCORE (log 10)

Figure S1. Microbial taxa and ARGs enriched in hospitals and river upstream samples. Linear discriminant analysis was used to identify both differentially enriched OTUs, based on 16S rRNA gene data (a, phylum, and b, genera), and ARGs (c). Upstream, grouped RS and RM samples; hospital, grouped hospital samples.

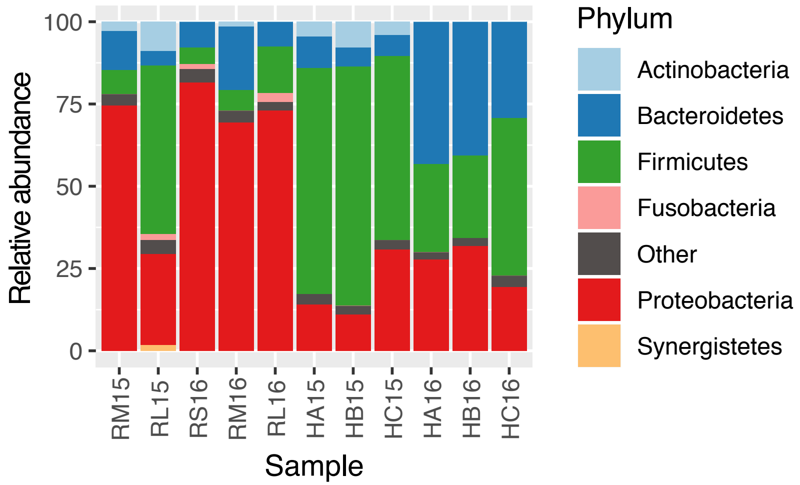

Relative abundance
100
75
50
25
0
RM15
RL15
RS16
RM16
RL16
HA15
HB15
HC15
HA16
HB16
HC16
Sample
Phylum
Actinobacteria
Bacteroidetes
Firmicutes
Fusobacteria
Other
Proteobacteria
Synergistetes

Figure S2. Metaxa2 community profiles. Taxonomic profiles determined with Metaxa2 coincide with those obtained with the analysis of 16S rRNA gene sequences (Fig. 2a).

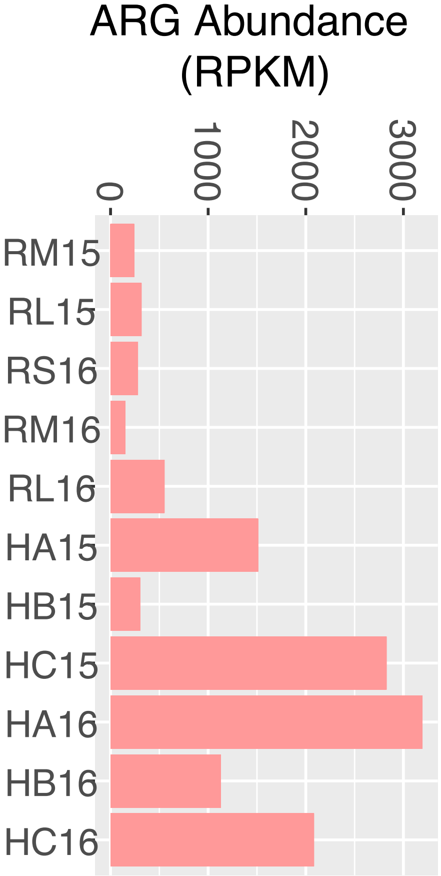

ARG Abundance
(RPKM)
0
1000
2000
3000
RM15
RL15
RS16
RM16
RL16
HA15
HB15
HC15
HA16
HB16
HC16

Figure S3. Abundance of clinically relevant ARGs and genetic determinants. Counts of clinically relevant ARGs and genetic determinants follow a similar pattern to general ARG counts (Fig. 4). The following were considered clinically relevant ARGs and genetic determinants: *AAC, aph, blaCTX-M, blaTEM, blaKPC, blaNDM-1, blaVIM, blaZ, cfr, CTX-M, defB, Erm, fmt, intI1, KPC, mecA, NDM, qnr, SHV, sul, TEM, tetM, vanA* and *VIM*.

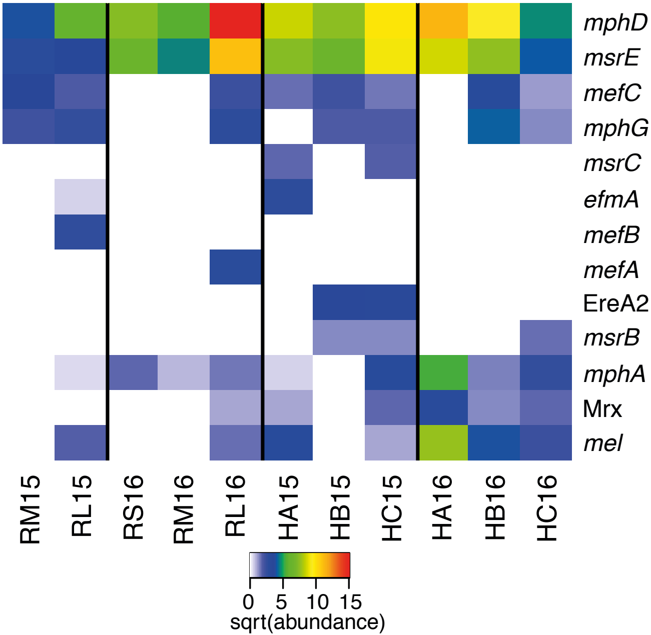
mphD
msrE
mefC
mphG
msrC
efmA
mefB
mefA
EreA2
msrB
mphA
Mrx
mel
RM15
RL15
RS16
RM16
RL16
HA15
HB15
HC15
HA16
HB16
HC16
0
5
10
15
sqrt(abundance)

Figure S4. Abundance of macrolide resistance genes. Macrolide resistance is present in all samples, but genes differ among samples.

Table S1
Drug concentrations in water samples.

	Drug concentration (µg/L)[b]						
**Sample[a]**	**CBZ**	**VEN**	**LOS**	**IRB**	**VAL**	**NAP**	**DIC**
RS15	ND	ND	ND	ND	ND	ND	ND
RM15	ND	ND	ND	0.01	0.12	0.14	0.03
RL15	0.04	0.01	3.24	0.08	0.62	0.67	0.32
HA15-1	ND	0.02	4.65	0.17	0.04	0.12	0.14
HA15-2	ND	ND	3.33	ND	0.83	0.38	0.19
HB15-1	0.37	ND	5.20	ND	ND	0.75	0.01
HB15-2	0.13	ND	4.59	ND	ND	0.35	0.09
HC15-1	0.14	0.03	6.42	0.77	38.42	0.38	ND
HC15-2	0.04	0.06	12.42	0.41	2.39	0.67	3.41

[a] Samples are labeled as in Table 1. No measurements were performed on the third replicate from the different hospitals.
[b] Measurements based on UHPLC-MS/MS. CBZ, carbamazepine; VEN, venlafaxine; LOS, losartan; IRB, irbesartan; VAL, valsartan; NAP, naproxen; DIC, diclofenac. ND: Not detected.

Table S2
Linear discriminant analysis of bacterial phyla in hospital wastewaters and upstream river locations.

Phylum	Category	Effect size	*p* value
Actinobacteria			-
Bacteroidetes			-
Cyanobacteria	Upstream	5.235159859	0.020136752
Firmicutes	Hospital	5.333049552	0.020136752
Fusobacteria	Upstream	5.036613241	0.020136752
Proteobacteria	Upstream	5.499567961	0.020136752
Synergistetes			-
Verrucomicrobia			-

Table S3
Linear discriminant analysis of bacterial genera in hospital wastewaters and upstream river locations.

Genus	Category	Effect size	*p* value
Acinetobacter			-
Aeromonadaceae, u. g.[1]			-
Akkermansia	Hospital	3.545583418	0.038867104
Alcaligenaceae, u. g.	Upstream	3.469430649	0.018102455
Allochromatium	Upstream	4.130821754	0.005761163
Aquabacterium	Upstream	4.167369558	0.020136752
Arcobacter			-
ASSO 13, u. g.	Upstream	3.555370551	0.020136752
Bacteroidales, u. g.	Upstream	4.159850902	0.020136752
Bacteroidales, u. g.	Hospital	4.915201262	0.020136752
Bacteroides	Hospital	4.317245333	0.038867104
Bifidobacterium			-
Blautia			-
C39	Upstream	4.551910355	0.015219242
Catenibacterium	Hospital	3.697349043	0.020136752
Christensenellaceae, u. g.	Hospital	3.642192588	0.020136752
Cloacibacterium	Upstream	4.072543894	0.020136752
Clostridiaceae_*Clostridium*			-
Clostridiales, u. g.	Hospital	3.586484169	0.020136752
Clostridiales, u. g.	Hospital	3.685965035	0.020136752
Collinsella			-
Comamonadaceae, u. g.	Upstream	4.367756338	0.020136752
Comamonas			-
Coprococcus	Hospital	3.721846755	0.020136752
Coriobacteriaceae, u. g.			-
Dechloromonas	Upstream	4.166762175	0.020136752
Desulfovibrio			-
Dialister	Hospital	3.960248409	0.020136752
Dorea	Hospital	3.733932183	0.020136752
Enterococcus			-
Erysipelotrichaceae, u. g.			-
Escherichia			-
Eubacterium			-
Faecalibacterium	Hospital	4.667492005	0.020136752
Flavobacterium	Upstream	4.132308723	0.020136752
Fusobacteriales, u. g.	Upstream	3.711860988	0.019620167

Hydrogenophaga	Upstream	4.317860841	0.018102455
Klebsiella			-
Lachnobacterium	Hospital	3.481667302	0.020136752
Lachnospira	Hospital	3.631495287	0.020136752
Lachnospiraceae_*Clostridium*	Hospital	3.452820384	0.020136752
Lachnospiraceae, u. g.	Hospital	3.542836689	0.020136752
Lachnospiraceae, u. g.	Hospital	4.261758899	0.020136752
Lactobacillales, u. g.			-
Lactobacillus			-
Lactococcus			-
Leptotrichia	Upstream	3.404820118	0.020136752
Limnohabitans	Upstream	4.84874854	0.020136752
Megamonas			-
Megasphaera	Hospital	3.648427688	0.020136752
Methylocaldum	Upstream	3.737013854	0.005761163
Methylophilaceae, u. g.			-
Methylosinus	Upstream	3.566029531	0.005761163
Microvirgula			-
Mitsuokella	Hospital	3.629537147	0.020136752
Mogibacteriaceae, u. g.			-
Neisseriaceae, u. g.			-
Novosphingobium	Upstream	4.044293742	0.020136752
Oscillospira	Hospital	3.98698583	0.020136752
Oxalobacteraceae, u. g.	Upstream	3.492188062	0.015219242
Paludibacter	Upstream	3.917087515	0.018102455
Parabacteroides			-
Peptostreptococcaceae_Clostridium			-
Peptostreptococcaceae, u. g.			-
Phascolarctobacterium	Hospital	3.848577001	0.020136752
Polynucleobacter	Upstream	4.564570633	0.005761163
Porphyromonadaceae, u. g.	Upstream	3.536438981	0.020136752
Prevotella	Hospital	3.447691179	0.020136752
Pseudomonas	Upstream	3.749935098	0.020136752
Rhizobiales, u. g.	Upstream	3.513716144	0.020136752
Rhodobacter	Upstream	3.904064787	0.019620167
Rhodoferax	Upstream	4.057502904	0.020136752
Roseburia	Hospital	4.385648447	0.020136752
Ruminococcaceae, u. g.	Hospital	3.930954563	0.020136752
Ruminococcaceae, u. g.	Hospital	3.817230618	0.020136752
Ruminococcus	Hospital	4.078853046	0.020136752

Lachnospiraceae, u. g.	Hospital	4.184708359	0.020136752
Selenomonas			-
SMB53			-
Streptococcus			-
Succinivibrio			-
Sutterella			-
Thiocapsa	Upstream	3.839333086	0.005761163
Tolumonas			-
vadinCA02			-
Veillonellaceae, u. g.			-
Zoogloea	Upstream	3.703766374	0.038055921

[1] u. g.: undetermined genus

Table S4
Classification of 16S gene copies identified by Metaxa2 in the metagenomic reads.

Sample[a]	Archaea (%)	Bacteria (%)	Eukaryota (%)	Uncertain (%)
RS16	0.27	80.70	12.01	7.02
RM15	0.32	85.88	8.20	5.60
RM16	0.20	87.68	6.51	5.61
RL15	1.80	96.21	0.39	1.59
RL16	0.26	98.19	0.38	1.16
HA15	0.03	98.06	0.19	1.72
HA16	0.00	98.43	0.24	1.32
HB15	0.04	97.99	0.06	1.91
HB16	0.00	98.18	0.29	1.52
HC15	0.04	98.44	0.08	1.45
HC16	0.04	98.89	0.08	0.99

[a] Sample names as described in Table 1.

Table S5
Linear discriminant analysis of antibiotic resistances in hospital wastewaters and upstream river locations.

Antibiotic	Category	Effect size	*p* value
Aminocoumarin			-
Aminoglycoside			-
Aminoglycoside_Fluoroquinolone			-
Bacitracin			-
Beta_lactam			-
Chloramphenicol			-
Fluoroquinolone			-
Fosfomycin			-
Glycopeptide			-
Lincosamide	Hospital	3.732079866	0.043114447
Lincosamide_Streptogramin			-
Macrolide	Upstream	4.505365933	0.020136752
Macrolide_Fluoroquinolone			-
Macrolide_Streptogramin			-
MicrocinJ25			-
MLS			-
Multiple			-
Mupirocin			-
Polymyxin	Hospital	3,671296553	0.043114447
Rifampicin	Hospital	3.608713849	0.043114447
Rifampicin_Isoniazid			-
Streptogramin	Hospital	3.793757722	0.043114447
Streptothricin			-
Sulfonamide			-
Tetracycline			-
Trimethoprim			-

Table S6
Linear discriminant analysis of ARGs in hospital wastewaters and upstream river locations.

ARG	Category	Effect size	*p* value
AAC(3)-Ia			-
AAC(3)-IIa			-
AAC(3)-VIIa			-
AAC(6)-30/AAC(6)-Ib			-
AAC(6)-Ib			-
AAC(6)-Ib-cr			-
AAC(6)-Ib'	Hospital	4.213581113	0.018102455
AAC(6)-Ib11	Hospital	3.354489843	0.043114447
AAC(6)-Ib3			-
AAC(6)-Ii			-
AAC(6)-IIa			-
aad(6)			-
aadA11			-
aadA12			-
aadA16			-
aadA2			-
aadA21			-
aadA23			-
aadA24	Hospital	3.931813751	0.018102455
aadA25			-
aadA5			-
aadA8			-
acrA			
acrD			-
acrE			-
acrF			-
acrS			-
aminocoumarin1			-
aminocoumarin2			-
amrA			-
ANT(2)-Ia	Hospital	3.259032123	0.043114447
ANT(6)-Ia			-
ANT(6)-Ib	Hospital	3.599819859	0.018102455
ANT(9)-Ia			-
APH(2)-IIa			-
APH(2)-IVa			-

APH(3)-Ia			-
APH(3)-Ib			-
APH(3)-IIa			-
APH(3)-IIIa	Hospital	3.177372808	0.043114447
APH(3)-VI			-
APH(6)-Id	Upstream	3.571471398	0.038867104
arr-2			-
arr-8			-
arnA	Hospital	3.283044964	0.043114447
bacA			-
baeR			-
baeS			-
Bifidobacteria			-
BlaB			-
bleomycin			-
catB2			-
catB3			-
catB8			-
catQ			-
catS			-
CblA-1			-
CcrA			-
CFE-1			-
CfxA			-
CfxA2			-
CfxA5	Hospital	4.072160589	0.018102455
CfxA6	Hospital	3.764412422	0.018102455
CMY-12			-
CMY-17			-
CMY-73			-
cphA5			-
cpxA			-
cpxR			-
CRP			-
CTX-M-101			-
CTX-M-107			-
CTX-M-11			-
CTX-M-117			-
CTX-M-12			-
CTX-M-72			-

CTX-M-9			-
dfrA12			-
dfrA14			-
dfrA15			-
dfrA17			-
dfrA19			-
dfrA21			-
dfrA8			-
dfrF			-
dfrG			-
efmA			-
efpA			-
emrA			-
emrB			-
emrD			-
emrK	Hospital	3.567456028	0.043114447
emrR			-
emrY			-
Enterobacter			-
EreA2			-
Erm(33)			-
ErmB	Hospital	3.762504796	0.018102455
ErmF			-
ErmG-1			-
ErmG-2			-
ErmT			-
Escherichia1			-
Escherichia2			-
evgA			-
evgS			-
floR			-
FosA3			-
FosA5			-
FOX-8			-
gadE			-
gadW			-
gadX			-
GES-1			-
GES-10			-
GES-21			-

GES-8			-
H-NS	Hospital	3.236558255	0.043114447
IMP-18			-
kdpE			-
Klebsiella			-
KPC-1	Hospital	4.1163439	0.043114447
KPC-11	Hospital	3.816688983	0.043114447
KPC-13			-
KPC-15	Hospital	3,835313675	0.043114447
KPC-22			-
KPC-8			-
KPC-9			-
LEN-13			-
leuO			-
lnuB			-
lnuC	Hospital	3.435178947	0.043114447
lnuF			-
lsaA			-
lsaC			-
lsaE			-
marA			-
mdtA			-
mdtB			-
mdtC			-
mdtD			-
mdtE			-
mdtF			-
mdtG			-
mdtH	Hospital	3.075169582	0.043114447
mdtL			-
mdtM			-
mdtN			-
mdtO			-
mdtP			-
mecR1			-
mefA			-
mefB			-
mefC			-
mel	Hospital	3.320631113	0.043114447
mfd			-

MOX-6			-
MOX-8			-
mphA			-
mphD	Upstream	4.382352889	0.020136752
mphG			-
Mrx	Hospital	3.114806755	0.043114447
msbA			-
msrB			-
msrC			-
msrE	Upstream	4.205195758	0.038867104
NDM-10			-
NDM-12			-
NDM-9			-
NPS			-
oqxA			-
OXA-119			-
OXA-129			-
OXA-14			-
OXA-15			-
OXA-164			-
OXA-17			-
OXA-170			-
OXA-183			-
OXA-2			-
OXA-210			-
OXA-233			-
OXA-239			-
OXA-25			-
OXA-253			-
OXA-27			-
OXA-31			-
OXA-333			-
OXA-334			-
OXA-34			-
OXA-36			-
OXA-36-2			-
OXA-361			-
OXA-368			-
OXA-370			-
OXA-398			-

OXA-415			-
OXA-420			-
OXA-422			-
OXA-454			-
OXA-46			-
OXA-47	Hospital	3.43089979	0.043114447
OXA-49			-
OXA-53			-
OXA-56			-
OXA-9			-
OXA-96			-
patA			-
PC1			-
PER-3			-
plasmid			-
PmrC			-
PmrE			-
qacH			-
QnrB21			-
QnrB22	Hospital	3.335935363	0.043114447
QnrB3			-
QnrB30			-
QnrB4			-
QnrB40			-
QnrB50			-
QnrB66			-
QnrB71			-
QnrB72			-
QnrD1			-
QnrS2			-
QnrS8			-
QnrS9			-
QnrVC1			-
sat-1			-
sat-4			-
SHV-115			-
SHV-135			-
SHV-159			-
SHV-167			-
SHV-29			-

SHV-34			-
sul1			-
sul2			-
sul3			-
TEM-10			-
TEM-101	Hospital	3.992049989	0.043114447
TEM-102			-
TEM-103			-
TEM-106	Hospital	3.876092602	0.043114447
TEM-108-1			-
TEM-108-2			-
TEM-112			-
TEM-113			-
TEM-123			-
TEM-125-1			-
TEM-125-2			-
TEM-127			-
TEM-135			-
TEM-138			-
TEM-145			-
TEM-146			-
TEM-148			-
TEM-149-1			-
TEM-149-2			-
TEM-150			-
TEM-156			-
TEM-159			-
TEM-164			-
TEM-166			-
TEM-172			-
TEM-174			-
TEM-178			-
TEM-182			-
TEM-185			-
TEM-189			-
TEM-192			-
TEM-196			-
TEM-208-1			-
TEM-208-2			-
TEM-22			-

TEM-54-1			-
TEM-54-2			-
TEM-55-1			-
TEM-55-2			-
TEM-57-1			-
TEM-57-2			-
TEM-59-1	Hospital	3.759509149	0.043114447
TEM-59-2	Hospital	3.956521022	0.043114447
TEM-89			-
TEM-95			-
tet31			-
tet32			-
tet39	Upstream	4,081712902	0.020136752
tet40	Hospital	3.572407771	0.043114447
tet44			-
tetA(P)			-
tetB			-
tetC	Upstream	3.687651667	0.020136752
tetD			-
tetE			-
tetL			-
tetM			-
tetO			-
tetQ	Hospital	4.072056258	0.018102455
tetS			-
tetT			-
tetW			-
tetX			-
tolC			-
vanA			-
vanHA			-
vanSA			-
vanSB			-
vanXA			-
vatE			-
VEB-9			-
vgaC	Hospital	3.357706818	0.043114447
VIM-23			-
YojI			-
